# Effects of a Peer-Tutorial Reading Racetrack on Word Fluency of Secondary Students With Learning Disabilities and Emotional Behavioral Disorders

**DOI:** 10.3389/fpsyg.2021.671385

**Published:** 2021-04-15

**Authors:** Anne Barwasser, Karolina Urton, Matthias Grünke

**Affiliations:** ^1^Department of Special Education and Rehabilitation, University of Cologne, Cologne, Germany; ^2^Department of Educational Sciences, University of Muenster, Muenster, Germany

**Keywords:** reading fluency, gamification, reading racetracks, peer tutoring, learning and behavioral problems, secondary students

## Abstract

Reading difficulties that are not addressed at the primary level continue to exist at the secondary level with serious consequences. Thus, it is important to provide struggling students with specific reading support. In particular, many students with learning disabilities (LD) and emotional behavioral disorders (EBD) demonstrate reading obstacles and are at risk for motivation loss. A multiple baseline design was used to evaluate the effects of a motivational reading racetrack as peer-tutoring on the word reading skills of secondary students with LD with and without EBD. The intervention was conducted through 4–5 baseline and 16–18 reading units three times a week for 15 min over 8 weeks. The results showed positive effects indicating a highly effective treatment. In addition, follow-up results were also promising. Our findings indicate that this multicomponent intervention has a positive effect on the word fluidity of low-achieving students in secondary education with LD and/or EBD.

## Introduction

### The Importance of Reading at the Secondary Level

Difficulties in reading at the secondary level are considered more serious than reading challenges at the primary level (Guerin and Murphy, 2015). Yet, the training of reading fluency is mainly carried out in the lower classes, as it is assumed that this is one of the tasks of primary school teachers (Rasinski et al., 2009). Thus, the promotion of reading at secondary level is often neglected (Edmonds et al., 2009). As a result, students with reading difficulties move further and further away from their typically performing peers, with the result that many fail to meet the requirements for each grade level. A recent edition of the Program for International Student Assessment (PISA) revealed that compared to the PISA survey in 2015, the reading performance of German youth had worsened (European Commission, 2018). Specifically, 21% performed below level 2 in reading which can be seen as high. Also, the survey showed that struggling German 15-year-olds did not enjoy reading as much as youth in other countries [Organisation for Economic Cooperation and Development (ECD), 2019]. Acquiring the reading skills necessary to become successful far beyond school is a major challenge for many students. Reading proficiency requires many complex steps. For example, lower-level processing skills such as decoding and reading fluency are necessary in order to advance toward higher-level skills such as reading comprehension (Chard et al., 2002; Kim et al., 2011).

### Hurdles in Achieving Reading Proficiency

On the road to reading proficiency, fluency is extremely important as it functions as a bridge between decoding and understanding a certain text; thus, without fluency, working memory (WM) capacities are used to simply decode a text, leaving little effort left to spend on attention to content (Juffs and Harrington, 2011) and, consequently, poorer comprehension. While stronger readers do decoding and vocabulary retrieval automatically *via* long-term memory, weaker readers have to consume more WM resources to improve reading, and especially sight word reading (Sweller, 1994; Peng et al., 2018). A meta-analysis by Peng et al. (2018) found a moderate relation between WM and reading (*r* = 0.29). Specifically, WM and word recognition were more strongly related than WM and non-word reading, and WM was more related to word reading than sentence reading. Barriers in reading fluency arise primarily from poor automation of reading sight words, resulting in poor mastery of decoding skills (Ayala and O’Connor, 2013). Deficits in automation in word recognition, in turn, pose a tremendous challenge to reading comprehension (Perfetti and Stafura, 2014). Coltheart et al. (2001), in turn, proposed a “dual-route theory” with regard to reading acquisition consisting of a lexical route and a non-lexical route. Using the *lexical route* (orthographic decoding) words are accessed quickly, whereas the *non-lexical route* (phonological recoding) consists of decoding individual words to be read, making this a more arduous process. Students with hurdles in the area of learning tend to rely on the non-lexical route since they struggle with storing information properly and, as a result, experience challenges in retrieving information rapidly. But word recognition is needed in order to become a proficient reader and thus, needs to get early attention. For the German language, Knoepke et al. (2014) showed that skills on the lexical route predict text comprehension better than skills on the non-lexical route. This underlines the importance of promoting the lexical route. Moreover, for German, which tends to be one of the transparent orthographies, students with reading difficulties face hurdles especially in automated direct word recognition (Landerl and Wimmer, 2008).

Students who did not learn word recognition skills in the earlier grades will most likely have reading difficulties, not only in the higher grades but throughout adulthood as well (Leffingwell, 2016). Ehri (2005) developed a model that deals specifically with the lexical path and word recognition. This model consists of the following stages: pre-alphabetic, partial alphabetic, full alphabetic, and consolidated alphabetic phase, which describes the degree to which readers make memory connections between the written word and pronunciation. Automated consolidated words enable the reader to master reading by quickly and unconsciously retrieving a word from the mental lexicon *via* the lexical route (Ehri, 2005).

### Students With Learning Disabilities and Emotional Behavioral Disorders

The majority of students with learning disabilities (LD) demonstrate hurdles in reading (Lerner and Johns, 2011), primarily reading fluency (Chard et al., 2002), due to challenges with processing information. Further, many students lose motivation to read and learn, and, understandably, get frustration (Martin et al., 2008). These factors may explain the PISA results with respect to reading motivation among German youth mentioned earlier.

Students with emotional behavioral disorders (EBD) present a growing challenge within the school setting (Forness et al., 2012). Problem behavior often has a negative effect on students’ school careers (Nelson et al., 2004; Chow and Wehby, 2018), including a risk of kids dropping out of school (Bradley et al., 2008). Within the current context, students who face challenges with reading, spelling, and/or math often display inappropriate and aggressive behavior (Auerbach et al., 2008; Pierce et al., 2013). Additionally, it has been reported that students with behavioral issues have a higher risk of deficits in language compared to their peers without behavioral challenges, especially with respect to reading skills (Benner et al., 2002; McCabe and Meller, 2004; Hilsmer et al., 2016). A meta-analysis by Hollo et al. (2014) estimates that 81% of students with EBD have negative experiences with reading and writing that go unnoticed for a long time, as the main focus is on fostering appropriate behavior. Given the importance of reading literacy, the large number of underachieving secondary school students, and the high correlation between LD, EBD, and inadequate reading proficiency and decreasing motivation, an intervention that addresses all of these is critically important.

### Ways to Foster Reading Competency

#### Repeated Reading and Sight Word Training

In order to effectively combine the previous components and integrate them into a reading intervention, the method of repeated reading (RR) at the word level can be introduced as a core element. A synthesis by Stevens et al. (2017) revealed that RR interventions positively affected the reading fluency of students with LD. Moreover, small positive effects were also found with respect to comprehension. These findings concur with those of Chard et al. (2002) and support the use of drill-and-practice methods for automation. For example, in their study with sixth-grade students with LD and EBD, Escarpio and Barbetta (2016) found that when the students read the same material repeatedly and got feedback from a tutor, they were able to read more words per minute and performed better on a reading comprehension test.

The addition of the model of Ehri (2005) and the relevance of the lexical route help to make sight word training an effective option for improving reading proficiency. When teaching words, it is important to provide numerous opportunities to practice the specific words and give feedback. A meta-analysis by Scammacca et al. (2007) showed that older students with reading difficulties with and without LD (4th–12th graders) benefited from interventions that were focused on the word level. A follow-up meta-analysis by the authors (Scammacca et al., 2015) reached a similar conclusion, showing the benefit of reading training at the word level. Thus, both studies showed that children can benefit from reading support up to grade 12, making it particularly relevant for secondary school readers who face severe failure in reading.

#### Reading Racetracks

Repetitive sight-word reading can be embedded in a reading racetrack procedure. A racetrack consists of empty cells equipped with little flashcards including content such as phonemes, words, or mathematical exercises to be trained extensively (Erbey et al., 2011). While this procedure has been shown to be effective with second-language learners and students with diverse disabilities (Alexander et al., 2008; Hopewell et al., 2011; Grünke, 2019; Grünke and Barwasser, 2019; Sperling et al., 2019), to date, it has not been investigated with secondary school students with LD with and without EBD.

#### Peer Tutoring as a Tool for Inclusion

To make an intervention, an inclusive tool, peer-tutorial learning can be added by having weaker and stronger children practice together. In general, peer-tutoring procedures are known for having a beneficial influence when being embedded in interventions (Mercer et al., 2011). These results are supported by the review of Stenhoff and Lignugaris/Kraft (2007) for secondary students in heterogeneous peer-tutoring settings. Okilwa and Shelby’s (2010) literature synthesis points in the same direction by showing that peer tutoring effects academic achievement positive in a variety of subject areas for 6- to 12-year-olds regardless of their type of disability (learning disability, emotional or behavioral disability, and intellectual disability). This is also confirmed in the meta-analyses by Bowman-Perrott et al. (2013) and Moeyaert et al. (2021) for single case data, which show that peer-tutoring has an significant effect on both academic (see also McDuffie et al., 2009) and social-behavior outcomes. In the meta-analysis by Bowman-Perrott et al. (2013), those with emotional and behavioral disorders benefitted most whereas Moeyaert et al. (2021) revealed a slightly larger effect on academic outcomes. With regard to reading skills, a study by Calhoon (2005) found positive effects of peer tutoring with low-reading middle school students on phonological skills and reading comprehension, but not on reading fluency. However, it should be noted that reading fluency was not taught directly, suggesting that peer tutoring might be effective on reading fluency as part of reading fluency training. The results regarding reading comprehension for secondary students with disabilities were also confirmed by a review of Alzahrani and Leko (2018). In general, peer-procedures seem to be beneficial in secondary special education (King-Sears, 2021). Considering students with reading and behavioral problems, results show that when two students are working together in order to improve specific content, reading competency can be enhanced both for those with and without problem behavior (Bowman-Perrott et al., 2013).

#### The Advantages of Incorporating Motivational Components

Considering the findings of the PISA study in the context of motivation and the result that especially secondary school students with reading hurdles lose motivation and enjoyment in reading [Organisation for Economic Cooperation and Development (ECD), 2019], there is an urgent need for motivational reinforcers to transform the reading experience into a more positive one for many students.

#### Group Contingencies and Self-Graphing Procedures

Elements such as group contingencies (GC; rewards dependent on group performance) and self-monitoring have also been demonstrated to be beneficial in the classroom as a means of supporting learning. The use of amplification systems (Bowman-Perrott et al., 2013) or, more specifically, GC procedures (Slavin, 1995; Rohrbeck et al., 2003) are particularly effective. In the implementation of tutorial learning, use of the GC procedures is a key success factor. Thus, research results confirm that procedures in which GCs are implemented, on average, achieve better results in terms of learning outcomes (Slavin, 1995; Rohrbeck et al., 2003) and improved social skills (Ginsburg-Block et al., 2006). Especially, interdependent group contingencies (IGC) procedures, in particular, have been found to be predictors of the success of peer-supported learning (Rohrbeck et al., 2003; Ginsburg-Block et al., 2006). Thus, studies using amplifiers had significantly greater effects on learning gains (i.e., Rohrbeck et al., 2003). Rohrbeck et al. (2003) published significant effects of using group reward contingencies in peer interventions [*p* < 0.05, *g* = 0.34 (with GC); *g* = 0.26 (without GC)]. Further, Popkin and Skinner (2003) showed that the use of specifically IGC has a positive effect on performance in different areas.

Self-monitoring procedures, which are related to self-regulation, have also proven to be beneficial for increasing performance. For example, Richards et al. (1976) found that students who monitored themselves in reading had stronger performance gains than students who received training without self-monitoring. More recently, a study by Stotz et al. (2008) showed positive effects on the number of total written words and number of correct word sequences with the implementation of a self-graphing procedure. Finally, Menzies et al. (2009) suggested that self-graphing particularly for reading performance can have positive effects on motivation and engagement.

Apart from the need for motivational elements, the demand for effective instructional methods that can be implemented with a heterogeneous learning group is increasing, especially due to the increasing heterogeneity of today’s classrooms.

### Research Aim

Given the increasing number of struggling secondary school readers with LD with and without EBD and the resulting risk of loss in motivation, an intervention that has a positive effect on both reading and motivation is essential. To make such an intervention applicable to inclusive classrooms, and, therefore, appropriate for students with varying abilities, the addition of a peer-tutorial procedure would be helpful. To fill the research gap on the issue of sight-reading and secondary-level students, the present study investigated whether older students with challenges in learning and behavior could benefit from a combined racetrack intervention. Thus, the core research question of the study was as follows: Does an intervention consisting of peer-tutorial reading tracks with gamified components have a positive impact on the word recognition of struggling secondary school students with LD with or without EBD?

## Materials and Methods

### Participants and Setting

Participants were 16 students with LD and EBD in grades 5–7 attending a low social-economic German urban special needs school in North Rhine-Westphalia. First, consent forms were sent to the parents of prospective participants, and data were only collected on students whose parents had agreed to the survey. Subsequently, a German reading screening [Salzburger Reading Screening (SLS); Wimmer and Mayring, 2014] was used in a first step in all classes (5–7, *N* = 37) to identify students with a reading quotient (RQ) below 89 as a cutoff for lower reading performance.

With regard to the intervention, which was to include peer tutoring with struggling and more advanced readers, the students with a lower RQ (< 79) were selected as tutees and those with a higher RQ (> 100) as tutors. In order to compile the reading pairs, the values of the reading screening were ranked, and the rank was divided in the middle. The student with the lowest score on the first half was paired (low RQ) with the student with the lowest score on the second half (high RQ) according to Fuchs et al. (1997). Care was taken to ensure that the students in the pairs understood each other well, based on advice from the teachers.

Overall, however, the reading performance of the participating classes was below an RQ of 95. Thus, the overall reading performance fell in the lower range. The reading screening resulted in 18 participants (nine tutors and nine tutees). Only data on the tutees were collected because the tutors had to be able to read the words to be trained fluently on the racetrack in order to be eligible to participate. One participant was not included in the data analysis due to missing data; consequently, only data on eight tutees are shown in the following.

All eight tutees from whom data were collected had been diagnosed with LD. Four of the students also diagnosed with an EBD. In Germany, the diagnosis of LD is determined contingent on repeated serious school failure in several subjects and EBD can be defined in Germany as getting special educational support with the focus on emotional and social development when a student cannot be adequately supported at school due to behavioral difficulties and his or her own development or that of his or her classmates is significantly disturbed or endangered. Both, students with LD and/or EBD receive special needs support in schools. All participants were native speakers of German (Table 1).

**Table 1:** Characteristics of participants.

Participants	Gender	Age	IQ	Special needs	Reading proficiency (LQ)	German L1
John	Male	11;7	70–85	LD/EBD	78	Yes
Timo	Male	12;1	70–85	LD/EBD	<62	Yes
Emma	Female	12;5	70–85	LD/EBD	64	Yes
Levin	Male	11;7	70–85	LD	69	Yes
Ben	Male	12;9	70–85	LD	<62	Yes
Sam	Male	12;3	70–85	LD/EBD	<62	Yes
Seba	Male	14;3	70–85	LD	68	Yes
Lauren	Female	13;1	70–85	LD	<62	Yes

LD, learning disabilities; EBD, emotional behavioral disorder; L1, first language; LQ, reading quotient (<89 underdeveloped reading; <79 weak reading).

### Design

A multiple baseline design within an AB plan (Ledford and Gast, 2018) was implemented with a total of 24 planned measurement points and three different baseline lengths. The reason for using a multiple baseline design was the experimental control it provides by decreasing the probability of alternative explanations for intervention effects (Byiers et al., 2012). Each group was supervised by one female master’s level student of special needs education and was taken out of the classroom for both the baseline phase and the intervention phase and supported in extra rooms. Data were collected after baseline sessions and after each intervention session.

The students were randomly divided into three groups. The first small group had a baseline length of four sessions, the second group of five, and the third group of six, after which the intervention began directly for each group. Thus, John, Timo, and Emma started with a baseline length of four, Levin and Ben with a length of five, and the remaining three, Sam, Seba and Lauren, with six baseline sessions. In total, the groups were taken out three times a week over 8 weeks Monday, Wednesday, and Friday always at the same time. The follow-up measurements took place 4 weeks after the end of the intervention, 2 weeks of which were Christmas holidays.

### Dependent Variables and Data Collection Procedure

The measuring tool was a researcher-made PowerPoint presentation with a 30-slide word sequence, into which words that were to be read out for 1 s each were visibly inserted with one word per slide (Ehri, 2005). Data from each tutee were collected after each baseline and intervention session to evaluate the impact of the intervention and a possible correlation to increases in single word reading. The number of correct and incorrect words read out loud was recorded. A word was considered correctly read if the tutee read the word within the 1-s interval of its occurrence. A word was considered to be misread if the tutee either omitted part of the word, added something to the word, or read it incorrectly. If the student corrected him/herself before the next word appeared, the word read aloud was recorded as correct. At no point during the word test did the students receive any help or feedback. The training words were shown in a different order at each measurement (and also at follow-up). The measurements were carried out by master’s-level students in pairs to ensure impartiality, with an interrater reliability of 100%.

### Material

**Figure 1:**
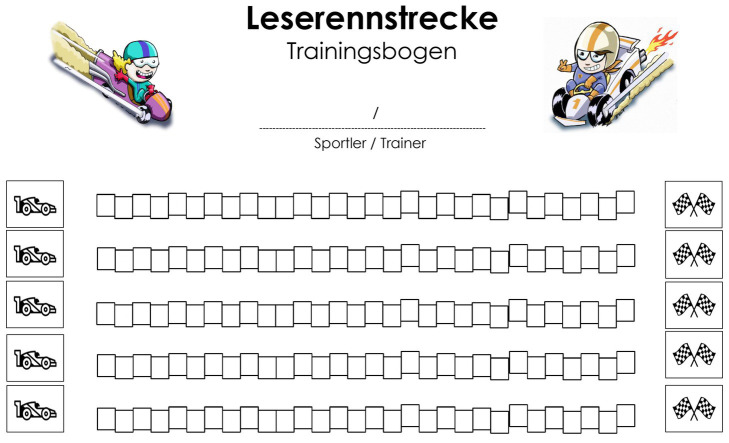
Self-graphing sheet. Leserennstrecke, reading racetracks; Trainingsbogen, training sheet; Sportler, athlete.

The material consisted of a playing field in A3 size (11.7 × 16.5 inches), which was divided into 30 fields and embedded in a reading racetrack. Each team received a small wooden figure (i.e., a race car or an animal) and a dice. In addition, the students were given 30 white laminated flashcards in an envelope. Each flashcard contained a different word. In order to find the respective words, a PowerPoint presentation (the same procedure used for the measurement) with 120 words was used before the start of the study to increase the probability of students finding 30 words that were not stored as sight words. The 120 words were two to four syllables long and reflected the most frequently occurring words in the German language. The selection of words was taken from a list published by the University of Leipzig.^1^ Care was taken to ensure that the words were of similar difficulty and did not exceed two or three syllables in length. From these 120 words, the final 30 training words for the racetrack were selected with a mean word frequency of 60.08. A stopwatch was used to measure time and a training sheet was used as a line chart to record the individual results of each team, as part of the reward system. The training sheet was comprised of 12 lines, listed one below the other. Each row, in turn, had 30 blank boxes for the maximum possible number of correctly read words per measurement (Figure 1).

### Procedures

#### Baseline

For the baseline condition, all students worked in their small groups and the assigned pairs in cognitive exercises, focusing mainly on sorting symbols into the correct order. The students were assigned as either tutee (low reading) and or tutor (more advanced reading). The length of the baseline condition was the same as the racetrack intervention in phase B (15 min). Subsequently, the measurement was performed individually for each subject. The groups were conducted at the same time in three different rooms.

#### Intervention

In phase B (reading racetracks), the participants practiced the 30 words selected from the previous word-tests repeatedly in the same group as well as in tutor/tutee team as in the baseline condition.

Prior to the start of the study, tutors were trained by the interventionists to provide feedback during the race game during a 1.5 h training session. Tutors were given example situations, with the task being how they would respond as a tutor, and training on how an adequate tutor would respond. The tutors were then divided into tandems, with one of the tutors taking the role of the tutee and both playing the racetrack game as an example. The tutees in the study were not present for this training.

At the beginning of the intervention, the previously selected tandems, consisting of one tutee and one tutor, sat down at a table where the 30 index cards were placed on the board with the printed word facing down. In the first sessions, all tandems were given an intensive explanation of the racecourse procedure and the roles of tutor/tutee as coach/athlete. The tutors’ role was to provide feedback and to correct if necessary and the tutee was asked to read around the racetrack. At the beginning, the tutees rolled the dice and moved their figure forward according to the number of points rolled. Then, the card was turned over and the word printed on it was read aloud. Meanwhile, the tutors listened carefully, corrected, if there were no self-correction by the tutees within 3 s, and repeated the word again correctly. If the word was correct, the tutors praised the tutees and the tutees went on with their figure on the game board. During reading, the index cards remained on the table with the word facing up. When all the words were read, the deck was reshuffled and the game started again. After 10 min, a signal indicated that the game was over. Measurements were then taken for each tutee individually.

As a reward system, the children recorded the number of words read correctly by the tutees on a self-graphing sheet after each measurement in phase B to document their own learning progress. A line of 30 quarters represents one session and the quarters represent the number of words read correctly per session. Depending on the score achieved, there was a reward in the form of marbles. Tutees received one marble if they achieved the same number of words read correctly as last time, and two marbles if they improved. The marbles were kept in a container. A group target was set in terms of the number of marbles in the container, so that the whole group received a reward as a group contingency procedure. The reward system was intended to increase student motivation (Kim et al., 2011).

### Treatment Fidelity

To ensure treatment fidelity, a checklist was designed to be completed by the master’s students after each session; in addition, for a third of the sessions, an external person filled out the questionnaire as well. The goal was to find out if the interventionists implemented the intervention as previously planned.

The checklist consisted of a table in which the subject codes were entered and whether they were present or not. Additional areas included “environment/framework conditions,” “material,” “course of support,” “diagnostics and feedback,” and “dealing with student behavior,” each with several items to be answered on a 5-point Likert scale from 0 = “does not apply at all” to 4 = “applies completely.” These areas were measured to ensure that the intervention was performed in exactly the same way in all three groups.

Before the study started, the first author gave a detailed briefing on the screening and conducting the baseline condition and intervention for 2 days in a row. In addition, a detailed guide was developed on how to conduct the study along with a time schedule. The first author was in regular weekly contact with the interventionists. The treatment fidelity agreement was 100%.

### Social Validity

To measure social validity, after the study participating students were asked to rate the following eight items with the help of a self-designed questionnaire using a 5-point Likert scale ranging from 0 (“totally disagree”) to 4 (“totally agree”).

1. The racetrack helped me to read words correctly.
2. I think the support also helps other students with reading difficulties.
3. I understood the meaning of the intervention well.
4. I learned a lot during the intervention.
5. I enjoyed coming to the intervention.
6. I would participate in the intervention again.
7. The words were difficult.
8. I enjoyed playing in pairs.

### Data Analysis

Analyses were conducted using the SCAN package for R by Wilbert and Lueke (2019). First, a visual inspection is performed, and the descriptive data are presented. For a more in-depth analysis, overlap measures will be used to determine the effectiveness of the intervention, and a level 2 regression analysis will be conducted across all subjects, focusing on the slope, the increase from the A phase to the B phase, and the level effect of whether there is a direct increase in the onset of the intervention. Within the overlap measures, we use the Non-Overlap of All Pairs (NAP; Parker et al., 2011a), the Percentage Exceeding the Median (PEM; Ma, 2006), the Percentage of All Non-Overlap data (PAND; Parker et al., 2007), and the Tau-U derived from Kendall’s rank correlation and Mann-Whitney *U* with possible A-phase trend correction (Parker et al., 2011b; A vs. B + TrendB − TrendA). The NAP is the percent improvement in data across phases, with 0.0–0.65 indicating a weak effect, 0.66–0.92 a moderate effect, and 0.93–1.0 a large effect. The PEM is the percentage of data points that exceed the median of the baseline. Less than 0.7 is a non-effective treatment, 0.7–0.9 is a moderate effect, and above 0.9 is a large effect. PAND is the total number of data points that do not overlap between phases, with individual data points not biased by outliers: 50–70% is a weak effect, 70–90% is a medium effect, and above 90% is a large effect. The Tau-U values can be divided into: up to 0.20 improvement can be considered as small change, 0.20 to 0.60 as moderate change, 0.60 to 0.80 as large change, and above 0.80 as very large change.

## Results

**Figure 2:**
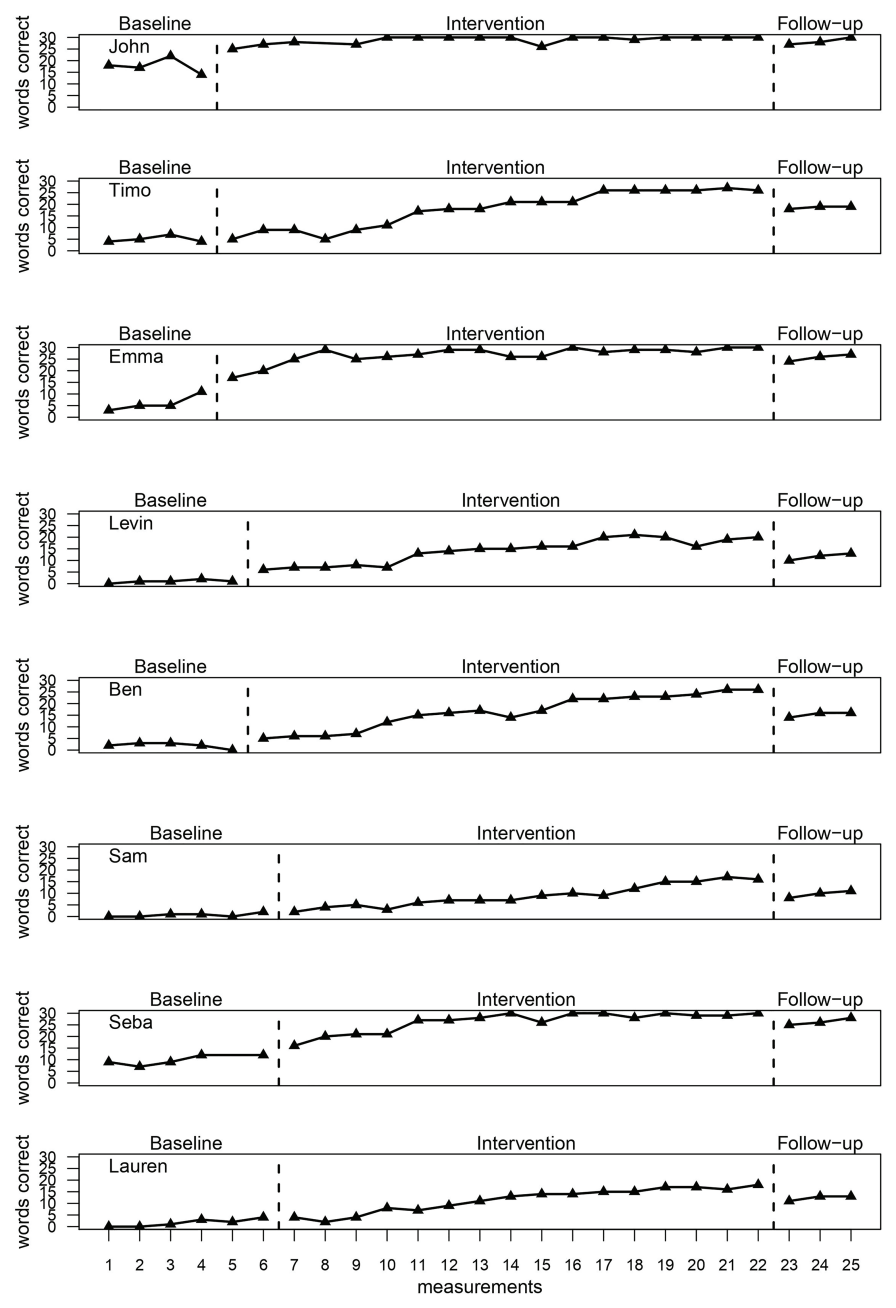
Amount of words read correctly.

Visually, it was clear that two participants, John and Seba, started with higher values in the baseline, with Seba stabilizing at the end and a downward trend for John. Possible positive baseline trends can be seen for Emma and Lauren. All other baselines appear to be low and flat. In phase B, a rapid increase in the number of correctly read words can be seen for all students, with some even showing a ceiling effect. The follow-up data can be described as relatively stable, with all probands showing a slight decline in value, but still well above the values for phase A (Figure 2; Table 2).

**Table 2:** Descriptive data for each participant in phases A, B, and E.

	*N* (A)	*N* (B)	*N* (E)	*M* (A) (*SD*)	*M* (B) (*SD*)	Max (B)	*M* (E) (*SD*)
John	4	18	3	17.75 (3.30)	28.94 (1.68)	30.00	28.33 (1.53)
Timo	4	18	3	5.00 (1.41)	17.83 (7.89)	27.00	18.67 (0.58)
Emma	4	18	3	6.00 (3.46)	26.83 (3.50)	30.00	25.67 (1.53)
Levin	5	17	3	1.00 (0.71)	14.12 (5.27)	21.00	11.67 (1.53)
Ben	5	17	3	2.00 (1.22)	16.53 (7.31)	26.00	15.33 (1.15)
Sam	6	16	3	0.67 (0.82)	9.00 (4.77)	17.00	9.67 (1.53)
Seba	6	16	3	9.80 (2.17)	26.38 (4.41)	30.00	26.33 (1.53)
Lauren	6	16	3	1.67 (1.63)	11.50 (5.19)	18.00	12.33 (1.15)

A, baseline; B, intervention; E, follow-up; N, measurements; M, mean; SD, standard deviation; Max, maximum value.

Overall, the average mean value in phases A, B, and E (follow-up) was 5.49, 18.90, and 18.50, respectively. This means that there was an overall increase of 1.790% from phase A to phase B. Three of the students achieved the maximum value of 30 during the intervention compared to the minimum value of 17 in phase B (Table 3).

**Table 3:** Overlap indices for the number of words read correctly comparing phases A and B.

Participants	NAP	*p*	PAND	Tau-U	*p*
John	100	<0.001	100	0.51	<0.001
Timo	92.00	<0.01	84.10	0.84	<0.001
Emma	100	<0.01	100	0.68	<0.001
Levin	100	<0.001	100	0.81	<0.001
Ben	100	<0.01	100	0.93	<0.001
Sam	99.00	<0.001	95.50	0.84	<0.001
Seba	100	<0.001	100	0.65	<0.001
Lauren	93.00	<0.001	90.90	0.80	<0.001

NAP, non-overlap of all pairs; PAND, percentage of all non-overlapping data.

Regarding the NAP, all students achieved high values, ranging from 99.00 to 100.00, except for Timo, who reached a value of 92.00. These results can be interpreted as statistically significant either at the < 0.01 level or < 0.001 level.

For the PAND, a mean effect of 84.10 was found for Timo and a high effect size with values from 90.90 to 100.00 for the rest of the sample.

Weighted Tau-U scores (A vs. B + trend B − trend A) showed a moderate effect for John (*p* < 0.001) and a large change for Lauren (*p* < 0.001), Seba (*p* < 0.001), and Emma (*p* < 0.001). For Timo, Levin, and Sam, a very large change was observed (*p* < 0.001). Furthermore, all results were statistically significant (Table 4).

**Table 4:** Regression model for the number of words read correctly (level-2-analysis).

	*B*	*SE*	*t*	*p*
Intercept	4.811	2.516	1.912	0.06
Trend	0.303	0.284	1.066	0.29
LevelB	4.454	0.920	4.841	<0.001
LevelE	4.524	5.663	0.799	0.43
SlopeB	0.596	0.288	2.073	<0.05
SlopeE	0.947	0.780	1.214	0.23

The regression analysis across all participants at level 2 displayed a statistically significant level effect from phase A to phase B (*p* < 0.001). A statistically significant slope effect with an average increase of 0.60 words read correctly per session was found when comparing the two phases (*p* < 0.05). Furthermore, no statistically significant difference was found for the E phase compared to the B phase.

### Social Validity

After the intervention, participating students were asked to complete the social validity questionnaire anonymously. Overall, they rated the intervention very positively on all issues. The highest score of *M* = 4.00 and an *SD* = 0, was given to items 2 (“I think the support also helps other students with reading difficulties”) and 8 (“I enjoyed playing in pairs”). This was immediately followed by items 1 (“the racetrack helped me to read words correctly”) and 5 (“I enjoyed coming to the intervention”) with a mean value of 3.88 and an *SD* = 0.33. Item 4 (“I learned a lot during the intervention”) received a mean score of 3.75 (*SD* = 0.43), item 3 (“I understood the meaning of the intervention well”), a mean value of 3.50 (*SD* = 0.25), and item 6 (“I would participate in the promotion again”), a mean value of 3.38 (*SD* = 0.48). Finally, responses to item 7 (“The words were difficult”) revealed that the training words were not too difficult for the students (*M* = 0.63, *SD* = 0).

## Discussion

The objective of this study was to evaluate the effects of a peer-tutorial reading racetrack intervention on the word fluency of secondary students with LD and those with a co-morbidity of LD and EBD. In line with other research (e.g., Hyde et al., 2009; Green et al., 2010; Erbey et al., 2011; Hopewell et al., 2011; Grünke, 2019), our results indicate that the reading racetrack intervention described in this paper was very effective in improving students’ ability to automate the reading of trained words. This also applies to the long-term effects. No significant decrease was evident here compared with the intervention effects for the group as a whole.

By applying the intervention at the secondary level and with students with LD, as well as students with LD and EBD, our study demonstrates that reading racetrack interventions can be used effectively with a heterogeneous student population. Further, while many previous studies have suggested that the intervention is effective in primary school (e.g., Grünke, 2019), the present study provides evidence that secondary students can benefit from word-level reading interventions as already shown in the meta-analyses by Scammacca et al. (2007, 2015). According to the meta-analysis by Hollo et al. (2014), particularly students with EBP have had numerous negative experiences in reading and writing, so it is important to balance them with positive learning situations. That this is feasible with the intervention described here is clearly demonstrated by the students’ assessments of social validity – the students viewed the intervention as both helpful and motivating. Also, the results go in line with previous studies and meta-analysis on the effects of peer-tutoring regarding students with disabilities (Stenhoff and Lignugaris/Kraft, 2007; Bowman-Perrott, 2009; McDuffie et al., 2009; Okilwa and Shelby, 2010; Alzahrani and Leko; 2018; Moeyaert et al., 2021). Moreover, these findings follow on from King-Sears (2021) that peer tutoring is generally well suited to secondary special education. Further, our study gives additional insights that reading fluency can be achieved through peer-tutoring when fluency is directly focused (see Calhoon, 2005).

### Limitations and Recommendations for Further Research

The results of the present study must be interpreted with some reservations. Despite its encouraging results for secondary students, the study is subject to the same weaknesses as all single-case designs, including a lack of generalizability due to the small sample size, which affects the external validity of the study. However, this circumstance can be compensated for by the evidence of previous studies showing the effectiveness of reading racetracks for the training of sight words in German schools (e.g., Grünke and Barwasser, 2019; Barwasser et al., 2021). Since the effectiveness of reading racetracks for students at higher grade levels has received little research attention so far, future studies should focus on this group of students in particular. Moreover, it has been shown that effect sizes with respect to peer-tutoring interventions are higher in quasi experimental designs and single group designs compared to randomized control trials indicating the fact that the stricter a research design is, the lower are the effect sizes (Zeneli et al., 2016). This is mainly due randomization of pre-tests which in turn control factors as, e.g., maturation and history threats (e.g., Trochim, 2012). This fact should be considered when interpreting the effect sizes displayed for this study. Additionally, further studies based on a randomized experimental-control group design should attempt to replicate the present results.

A further limitation of the present study is that we did not include a differentiated analysis of the students according to those with LD only and those with a co-morbidity of LD and EBD. Therefore, it might be of interest for further research to investigate differential effects in relation to the particular special educational needs of students. This is especially true for the long-term effects of the intervention. Although there was no significant overall decrease in the effects over time, the visual inspection for the individual students indicates that for some students, the competence level in the follow-up measurement decreased while it remained stable for others.

In addition to considering the tutees’ perspective on social validity, the tutors’ opinions also appear to be of central interest. The study by Vogel et al. (2007) gives an insight into the fact that the tutors were uncertain about dealing with learning difficulties and how to establish a good tutoring relationship, even though the interactions were rated as positive by both groups. Intensive training of the tutors could be considered here, which, in addition to teaching the content of the intervention, also clarifies the special support needs in learning of the tutees.

Finally, since the intervention consisted of several components (reading from the racetrack, motivational components peer tutoring), it is not possible to identify the specific effects of each element of the intervention. Therefore, it remains to be investigated in future research to what extent each of the components adds to the overall effectiveness. In order to draw conclusions about the extent to which the effects of the present study can be attributed not only to the practice activity of reading words itself but also to the intervention implemented, it remains important to conduct randomized experimental control group designs in future studies.

### Conclusion

In summary, our results confirm the effectiveness of a peer-tutorial reading racetrack intervention in promoting reading fluency for secondary students with LD and students with LD and EBD. Thus, the method has a wide range of application in terms of student age and special educational need. Given the small expenditure of materials and time makes this not only an effective but also an economic intervention for the classroom.

## Data Availability Statement

The raw data supporting the conclusions of this article will be made available by the authors for all interested researchers.

## Ethics Statement

Ethical review and approval was not required for the study on human participants in accordance with the local legislation and institutional requirements. Written informed consent to participate in this study was provided by the participants’ legal guardian/next of kin.

## Author Contributions

All authors listed have made a substantial, direct and intellectual contribution to the work, and approved it for publication.

### Conflict of Interest

The authors declare that the research was conducted in the absence of any commercial or financial relationships that could be construed as a potential conflict of interest.

## Footnotes

^1^https://wortschatz.uni-leipzig.de/de
